# Legionnaires’ Disease Outbreak on a Merchant Vessel, Indian Ocean, Australia, 2015

**DOI:** 10.3201/eid2407.171978

**Published:** 2018-07

**Authors:** Timothy J.J. Inglis, Chantal Spittle, Hilary Carmichael, Jaala Downes, Marilina Chiari, Adrian McQueen-Mason, Adam J. Merritt, Meredith Hodge, Ronan J. Murray, Gary K. Dowse

**Affiliations:** PathWest Laboratory Medicine WA, Nedlands, Western Australia, Australia (T.J.J. Inglis, M. Chiari, A. McQueen-Mason, A.J. Merritt, M. Hodge, R.J. Murray);; University of Western Australia, Crawley, Western Australia (T.J.J. Inglis, A.J. Merritt, M. Hodge, R.J. Murray);; Shire of Esperance, Esperance, Western Australia (C. Spittle);; Western Australia Country Health Service, Broome, Western Australia (H. Carmichael);; Western Australia Department of Health, Shenton Park, Western Australia (J. Downes, G.K. Dowse);; Sir Charles Gairdner Hospital, Nedlands (R.J. Murray)

**Keywords:** Legionnaires’ disease, Pontiac fever, outbreak investigation, merchant ship, vessel, cruise, *Legionella* *pneumophila*, bacteria, respiratory, environmental control, fomite, reservoir, biofilm, pneumonia, pneumothorax, PCR, deployable microbiology, health threat assessment, occupational risk, Indian Ocean, Australia

## Abstract

Two cases of Legionnaires’ disease and 1 of Pontiac fever occurred among the crew of a merchant ship operating off the shores of Australia. PCR assays identified potential sources in the ship’s cabins. Modification of maritime regulations for Legionnaires’ disease prevention in commercial vessels is needed for nonpassenger merchant ships.

The risk for Legionnaires’ disease (LD) is known on cruise liners (*1*–*3*) and is matched by recommendations for preventive measures (*4*,*5*). Environmental sources of *Legionella pneumophila* in ships are prone to transmit LD over several years through resistance to decontamination (*6*,*7*). As opposed to cruise liners, there are few reports of LD on working vessels, where occupational health risks differ (*8*). *Legionella* was detectable in potable water systems on 58% of 350 merchant vessels in a recent survey (*9*). There was no established precedent for environmental risk assessment or control when 2 LD cases occurred on a merchant ship off the northwestern Australian Indian Ocean coast in 2015. We therefore conducted an extended field investigation.

## The Study

The first LD case-patient on the merchant ship sought treatment at the nearest hospital emergency department, and provided no alternative exposure source. After laboratory confirmation of this case, the crew disembarked and the vessel was required to lie at anchor offshore. After emergency control measures by a private contractor, we obtained information on the ship’s plumbing, including potable, fresh, and hot water systems, water storage, air conditioning; food preparation areas, and sleeping quarters.

We then boarded the ship for environmental investigation on August 27, 2015, to collect samples from potential fomites around the vessel at 33 locations, including cabins and potable water outlets. We collected PCR swab samples in duplicate from inside showerheads and sink faucets (also known as mixer taps) aerators in sleeping quarters and food preparation areas, including those used by LD case-patients and their neighbors. The contractor disinfected the water system by using super chlorination the next day, and collected a second environmental sample series on September 4. Additional targeted control measures included replacement of showerheads and removal of faucet aerators from cabins.

**Figure 1:**
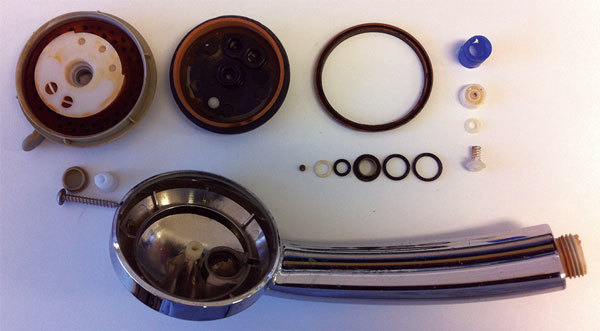
Dismantled showerhead from nonpassenger merchant vessel showing multiple inner parts, including 7 O-rings, all of which were in contact with water passing through shower, Australia, 2015.

We collected a series of PCR swab samples from original test locations on October 12 to assess the residual health threat, and tested 24 of these samples on the ship (*10*). Duplicate samples were then tested in the reference laboratory (*10*). We analyzed showerheads removed from cabins (Figure 1). We tested samples of the inside surface of each showerhead and its O-ring gaskets by using PCR assays. We collected swab samples from potential reservoirs and tested for *Legionella* species: the O-rings; rinse samples from showerhead parts in sterile 0.08% NaCl solution for *Legionella* species; peptone water washings, showerhead contents, debris from a thermal mixing valve, fresh and pre–UV-treated water, showerheads, air conditioners, and faucets from cabins (*11*). We identified presumptive *Legionella* cultures on MWY and BMPA agars by using *Legionella* Latex Agglutination antisera (Oxoid; ThermoFisher Scientific Australia Pty Ltd, Scoresby, Melbourne, Victoria, Australia), and cultured for amoeba on showerhead rinse specimens. Detailed methods are provided in Technical Appendix Part 1.

**Figure 2:**
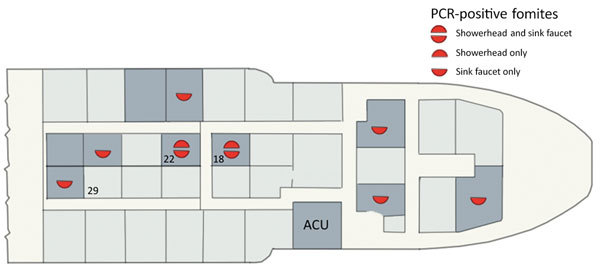
Accommodation deck plan, Australia, 2015. Cabins (n = 10) and other rooms (ACU, air conditioning unit) from which environmental samples were collected on August 27, 2015 are indicated in dark gray. PCR-positive locations are indicated by red semicircles; upper, shower water or swab; lower, mixer tap water or swab. The 3 case-patients occupied cabins 18, 22, and 29.

In August 2015, the Western Australia Department of Health was notified of Legionnaires’ disease confirmed by *L. pneumophila* serogroup 1 urinary antigen test in a member of the vessel’s crew (case-patient 1), and was informed that other crew members had mild febrile respiratory illness (Table 1). Later that day, another crew member, who had symptoms of severe bilateral pneumonia and pneumothorax, arrived at the regional hospital and required aeromedical evacuation for intensive care (case-patient 2). LD was confirmed by urinary antigen testing and PCR assay on bronchial washings. Other crew members who had nonpneumonic respiratory and other symptoms were investigated for legionellosis by using urinary antigen tests and serologic tests which proved negative, except in case-patient 3, who had *L. pneumophila* seroconversion and Pontiac fever that did not require hospital admission. The 3 cases all satisfied Australian LD case definitions (*12*). Case-patients 1 and 2 occupied adjacent cabins and case-patient 3 was 2 cabins away from case-patient 2 (Figure 2).

**Table 1:** Summary of confirmed legionellosis cases and results of environmental PCR testing in the case-patients’ merchant vessel cabins, August 2015*

Case-patient	Age, y	Onset	Infection	Hospital	UAT	Serology	PCR†	Cabin no.	Cabin samples (Aug 27)		
									Shower water	Shower-head swab	Bathroom sink faucet
1	54	Aug 12	Lower respiratory	Regional	+	_	+	22	+	_	+
2	55	Aug 19	Lower respiratory	Tertiary	+	_	+	18	+	+	+
3	48	Aug 10	Mild respiratory	Not required	_	Conversion (0–2,048)	_	29	NA‡	NA‡	NA‡

*NA, not available; UAT, urinary antigen test; +, positive; –-, negative. †PCR-positive *Legionella pneumophila.*  ‡Cabin in use on August 27, 2015. Water from hand basin faucet collected on August 20 by private agency was culture negative.

*L. pneumophila* was not isolated from any environmental samples. Legionella PCR result was positive in 7/10 cabins tested (13/27 samples) (Table 2). A PCR result was positive for showerheads or residual water from sink faucets in the cabins of 2 LD cases. In 5 other cabins, only faucets were positive (Figure 2). Detection of sludge or biofilm in the shower heads and faucets prompted replacement with better-designed showerheads and removal of faucet aerators. Only 2/79 samples collected on the second visit on September 4 were Legionella PCR positive; a significant reduction (χ^2^, Yates’ correction; 15.98, p<0.001). Only 1 of the 58 samples from the third series of samples was clearly PCR positive, from a faucet in a cabin unconnected to LD cases. The in-field PCR results were identical to the confirmatory reference laboratory replicate results. All 10 types of showerhead were rust-stained inside and smelled of chlorine. The most common showerhead types had either 7 silicone rubber O-rings or 1 complex silicone rubber gasket. Showerhead swabs and agar O-ring impressions grew profuse mixed bacteria, commonly *Pseudomonas aeruginosa*. Nonpneumophila *Legionella* sp. was isolated from 1 showerhead. Legionella PCR assays produced unambiguous positives in 13/16 showerheads (19/32 samples). Almost all O-rings from the common showerhead types were *Legionella* positive (Technical Appendix Part 2).

**Table 2:** *Legionella pneumophila* species PCR results from environmental samples collected on merchant vessel, Australia, 2015

Sample type	Samples collected on vessel, by date												Dismantled showerheads	
	August 27			September 4			October 12							
Reference laboratory testing		Reference laboratory testing		In-field testing			Reference laboratory testing		Reference laboratory testing	
Total	PCR+	Total	PCR+	Total	PCR+		Total	PCR+	Total	PCR+
Cabin shower heads	6	3		36	0		12	0		29	0		32	19
Cabin faucets	14	9		33	1		12	0		29	1		NA	NA
Air conditioning	4	0		NA	NA		NA	NA		NA	NA		NA	NA
Water supply	2	0		NA	NA		NA	NA		NA	NA		NA	NA
Others	1	1		10	1		NA	NA		NA	NA		NA	NA
Total results	27	13		79	2		24	0		58	1		32	19
PCR controls														
Positive, *Legionella* DNA extract	2	2		2	2		2	2		2	2		2	2
Negative, ultrapure water	6	0		16	0		5	0		12	0		6	0
*NA, not applicable; +, positive.														

A recent study of nonpassenger merchant vessels (NPMVs) highlighted the risk for *Legionella* contamination of potable water systems (*9*), but did not establish a link with confirmed infections. Our investigation of *L. pneumophila* serogroup 1 infection in a merchant vessel’s crew highlights the need to control *Legionella* in NPMV water systems, and the challenge of using PCR assays, which do not detect viable bacteria. Culture-dependent methods did not contribute to determination of the environmental source or route of dissemination. Preliminary control measures by external contractors may have prevented *Legionella* isolation from our environmental samples, but have doubtful long-term preventive value without sustained control measures because environmental persistence occurs in ships despite biocide treatment (*6*).

The survey vessel had a gross tonnage of 2,620, was 64 m long, 16 m wide, a draft of 4.7 m, and a crew of 27. It had 2 water storage tanks with 60,000 L capacity, an ultraviolet water sterilization unit, and 2 hot water geysers. These tanks were refilled from bunkers while in port, and replenished at sea by reverse osmosis. Showers were highlighted in a previous study of NPMV potable water systems (*9*), and aerator devices have been implicated as bacterial amplification sites in tropical and nosocomial outbreaks (*13*,*14*).

Multiple positive PCR results from water outlets in the cabins implicated the showers and faucets as means of infection. All showerheads on the vessel had interior moving parts to control spray settings and were the leading PCR-positive location. A rust-colored biofilm inside most showerheads indicated possible deterioration of iron pipes in the ship’s distribution system, and persistence of *Legionella* in biofilms (*15*). The silicone rubber O-rings from the showerheads supported profuse growth of aquatic bacteria and were PCR positive for *L. pneumophila*. The O-rings formed a permanently wet niche for bacterial growth, and their movement will shear bacteria from biofilms. Faucet aerators also promote turbulent flow by mixing water and air under pressure. These results highlight the potential for *Legionella* aerosol generation. We recommended replacing the showerheads with a simpler plastic design, more suited to periodic removal, decontamination, and cleaning, and gravity drainage after daily use.

## Conclusions

A cluster of *L. pneumophila* serogroup 1 infections in a vessel working in waters near Australia led to an environmental health assessment in which molecular methods enabled the field investigation team to implicate water outlets in crew quarters and tailor environmental controls. Deployment of quantitative PCR assays extended our investigative reach offshore, enabling faster return of the vessel to active service. The leadership and crew of nonpassenger merchant vessels operating in tropical waters need heightened *Legionella* awareness and require control measures more stringent than those applied in passenger vessels. 

Technical Appendix Detailed descriptions of environmental sample collection on the non-passenger merchant vessel at sea and in port, emergency control measures, and detailed results of PCR and culture to identify pathogens.

## References

[1] Centers for Disease Control and Prevention (CDC). Cruise-ship—associated Legionnaires disease, November 2003-May 2004. MMWR Morb Mortal Wkly Rep. 2005;54:1153–5. 16292248

[2] Azara A, Piana A, Sotgiu G, Dettori M, Deriu MG, Masia MD, et al. Prevalence study of *Legionella* spp. contamination in ferries and cruise ships. BMC Public Health. 2006;6:100. 10.1186/1471-2458-6-100 16620388 PMC1459133

[3] Goutziana G, Mouchtouri VA, Karanika M, Kavagias A, Stathakis NE, Gourgoulianis K, et al. *Legionella* species colonization of water distribution systems, pools and air conditioning systems in cruise ships and ferries. BMC Public Health. 2008;8:390. 10.1186/1471-2458-8-390 19025638 PMC2605755

[4] Mouchtouri VA, Rudge JW. Legionnaires’ disease in hotels and passenger ships: a systematic review of evidence, sources, and contributing factors. J Travel Med. 2015;22:325–37. 10.1111/jtm.12225 26220258

[5] Mouchtouri VA, Bartlett CL, Diskin A, Hadjichristodoulou C. Water Safety Plan on cruise ships: a promising tool to prevent waterborne diseases. Sci Total Environ. 2012;429:199–205. 10.1016/j.scitotenv.2012.04.018 22608187

[6] Ahlen C, Aas M, Krusnell J, Iversen O-J. A single *Legionella pneumophila* genotype in the freshwater system in a ship experiencing three separate outbreaks of legionellosis in 6 years. Microb Ecol Health Dis. 2016;27:31148. 27515183 10.3402/mehd.v27.31148 PMC4981653

[7] García MT, Baladrón B, Gil V, Tarancon ML, Vilasau A, Ibañez A, et al. Persistence of chlorine-sensitive *Legionella pneumophila* in hyperchlorinated installations. J Appl Microbiol. 2008;105:837–47. 10.1111/j.1365-2672.2008.03804.x 18557962

[8] Ahlén C, Aas M, Nor A, Wetteland PI, Johansen H, Sørbø T, et al. *Legionella pneumophila* in Norwegian naval vessels. Tidsskr Nor Laegeforen. 2013;133:1445–8. 23929291 10.4045/tidsskr.12.1459

[9] Collins SL, Stevenson D, Mentasti M, Shaw A, Johnson A, Crossley L, et al. High prevalence of Legionella in non-passenger merchant vessels. Epidemiol Infect. 2017;145:647–55. 10.1017/S0950268816002715 27890040 PMC9507734

[10] Lindsay DS, Abraham WH, Fallon RJ. Detection of mip gene by PCR for diagnosis of Legionnaires’ disease. J Clin Microbiol. 1994;32:3068–9. 7883904 10.1128/jcm.32.12.3068-3069.1994 PMC264231

[11] Standards Australia. Waters – Examination for *Legionella* species including *Legionella pneumophila*. Australia/New Zealand Standard Method 3896:2008. https://infostore.saiglobal.com/preview/as/as3000/3800/3896-2017.pdf?sku=1912444 [cited 10/12/2017]

[12] Legionellosis case definition. Department of Health, Government of Australia. http://www.health.gov.au/internet/main/publishing.nsf/Content/cda-surveil-nndss-casedefs-cd_legion.htm 12/20/2012 [cited 10/12/2017]

[13] Inglis TJ, Benson KA, O’Reilly L, Bradbury R, Hodge M, Speers D, et al. Emergence of multi-resistant *Pseudomonas aeruginosa* in a Western Australian hospital. J Hosp Infect. 2010;76:60–5. 10.1016/j.jhin.2010.01.026 20451300

[14] Inglis TJ, Garrow SC, Henderson M, Clair A, Sampson J, O’Reilly L, et al. *Burkholderia pseudomallei* traced to water treatment plant in Australia. Emerg Infect Dis. 2000;6:56–9. 10653571 10.3201/eid0601.000110 PMC2627980

[15] van der Lugt W, Euser SM, Bruin JP, Den Boer JW, Walker JT, Crespi S. Growth of *Legionella anisa* in a model drinking water system to evaluate different shower outlets and the impact of cast iron rust. Int J Hyg Environ Health. 2017;220:1295–308. 10.1016/j.ijheh.2017.08.005 28869187

