# Supplementary material for: Legionnaires’ Disease Outbreak on a Merchant Vessel, Indian Ocean, Australia, 2015
Source: Emerg Infect Dis. 2018 Jul;24(7):1345–8. doi: 10.3201/eid2407.171978 (PMC6038751; doi:10.3201/eid2407.171978)
Supplement: Technical Appendix — Detailed descriptions of environmental sample collection on the non-passenger merchant vessel at sea and in port, emergency control measures, and detailed results of PCR and culture to identify pathogens. [file 17-1978-Techapp-s1.pdf]

Article DOI: https://doi.org/10.3201/eid2407.171978

Legionnaires’ Disease Outbreak on a Merchant Vessel, Indian Ocean, Australia, 2015

Technical Appendix

Part 1: Detailed Methods

The investigation was conducted in accordance with the 2005 International Health Regulations (*1*,*2*).

Vessel Description, Water Storage, and Supply

Type: seismic survey vessel; tonnage 2,620 gross; 899 tons dead weight; extreme dimensions, 64 m long × 16 m wide, 4.7 m approximate draft; typical cruising speed 5.4–5.7 knots; crew, ≈ 27 with accommodation for up to 49.

Onboard Potable/Fresh Water Supply

At sea, reverse osmosis-treated seawater stored in two 60,000 L tanks, then passed through a UV disinfection unit before distribution to potable and hot water outlets. Two hot water geysers supplied water <60°C into separate ring mains. Ambient temperatures on board were 20–45°C and both hot and cold water distribution pipes were unlagged; conditions that could support *Legionella* growth.

In port (known as bunkering mode), potable water obtained from supply point on the jetty, and stored in two 60,000 L tanks before UV disinfection and distribution. Potable water distributed to 2 geysers in separate hot water circuits, set at 60°C in the geyser.

Legionnaires’ Disease Case Cluster

There were no common shore-based exposures linking the 3 confirmed Legionnaires’ Disease (LD) cases either immediately after disembarkation and the onset of symptoms or in the weeks before the arrival of the ship in the port in northwestern Australia.

Preliminary Investigation Planning

Conducted by teleconference and included the vessel's operators, senior crew, maritime safety agency, Kimberley Population Health Unit and Department of Health disease control and public health laboratory representatives. Information obtained on the on-board plumbing, potable and fresh water systems, water storage, air conditioning, catering and sleeping quarters, and planned deployment of a site inspection and environmental sample collection team.

Additional Emergency Control Measures

August 28, 2015

Disinfection/super-chlorination of on-board potable/fresh water system, as per AS/NZS 3500.1 and World Health Organization Guide to Ship Sanitation: Sodium hypochlorite dosed into water storage tanks to obtain free Chlorine residual of >20 mg/L, allowed a contact time of 40 min., then flushed through the potable/fresh water system and left for a contact time of >3h. Water storage tank contents were then neutralized with sodium thiosulfate and tanks and water outlets flushed to remove chlorinated water. Showerheads were removed and disinfected by immersion in chlorinated water. Further advice on specific sustainment control measures was provided by consultants recommended to the ship's operator.

Sample Collection Details

PCR swabs (first series, 27 August 2015) collected from 33 locations around the vessel, of which 27 were specifically for PCR. Location included washbasins (also called sinks), and showers in cabins and other potable water outlets such as in the galley and mess. Cabins were equipped with washbasins that had mixer taps, and shower water temperature was manually controlled by the user. Duplicate PCR swab specimens were collected from inside showerheads and washbasin mixer tap outlets through the sleeping quarters, including cabins used by confirmed LD case-patients and their neighbors (Figure 2). Disinfection (superchlorination) was performed the next day, and a second environmental sample series was collected 6 days later. An additional series of PCR swab specimens was collected from previously tested locations. Twenty-four of these samples were tested on board the ship using

our field deployable version of the reference laboratory PCR method to assess the residual health threat to the crew and with 24 duplicate samples from 12 cabins were subsequently tested in the reference laboratory.

Water samples: small volume (<5.0 mL) water samples were collected in cabin outlets and adjacent parts of the potable water system where water accumulated, or stagnated. The hot water supply was not accessed other than via final outlets (e.g., mixer taps).

Air samples; a compact impinger air sampler (MAS-100 Eco, Merck, Bayswater, Australia) was used to collect 1, 000 L air in shower cubicles in the cabins of LD case-patients and other locations on the vessel prone to fresh water aerosol generation. This used an Anderson sampler principle to draw 200 L of air at a constant rate onto a 9-cm diameter agar plate. One *Legionella* selective media (BCYE) plate and one hemolyzed blood (chocolate) agar plate was used at each location (i.e., two ×x 200-L air samples). These plates commenced incubation immediately after return to Perth on the same day.

Showerheads removed from cabins at the time of the initial environmental investigation were sent to the public health laboratory for additional analysis. The inside surface of the showerhead its O rings were tested by PCR assay.

• Mixer tap aerators were unscrewed and swabs used to sample the upstream surface

• The showerhead parts were rinsed with sterile 0.08% NaCl solution and the washings used to recover *Legionella* species and free-living amoebae.

PCR Methods

Swabs were eluted in 1 mL of Hanks balanced salt solution transport medium. DNA extraction was by magnetic beads. (MagNA Pure 96 DNA and Viral NA Small volume Kit and MagNA Pure 96 Pathogen Universal Protocol, Roche, North Ryde, Australia). Water samples were processed on the same platform without modification.

Identification of *L. pneumophila* was by a real-time PCR assay. The gene targeted was the macrophage infectivity potentiator gene (*mip)* of *Legionella species* (*3*). Primer and probe sequences used were as published (*4*). PCR master mix composition was as follows; 1× Quanta PerfeCTa qPCR Tough Mix and oligonucleotides at 0.2 µmol/L. Reaction volume was 20 µL and included 8 µL of template. Amplification was performed on RotorgeneQ thermal cyclers (Qiagen) using the following program; pre-PCR of 2 min at 95°C to fully

denature the template DNA and activate the polymerase followed by 50 cycles for 12 s at 95°C for denaturation, 15 s at 55°C for annealing and 20 s at 72°C for extension and fluorescence acquisition. Analysis was performed using a threshold of 0.05. Signals crossing the threshold before 40 cycles were considered positive detections. Some cross-reactivity of the assay with other *Legionella* species (notably *L. fairfieldensis* and *L. worsleiensis*) and inability to differentiate live and dead bacteria are known limitations of this approach. However, molecular detection of other *Legionella* species also indicates a Legionella-supportive environment.

Field Deployable PCR Assay

The primers and probes used were as above, assembled as a master mix dispensed in 1-mL aliquots and kept frozen during transport into location. The thermocycler used was a 16 well solid state cycler (MyGo Mini, IT-IS Life Science Ltd, Mahon, Republic of Ireland), allowing analysis of 12 sample and 4 control tubes. Our operation of portable thermocyclers in field conditions for bioreconnaissance and other environmental health threat applications has been reported and reviewed previously (5–9).

Water Sample and Outlet Fitting Cultures

After dismantling showerheads and collecting PCR swab specimens from the inside surface of the shower rose and any O-rings found, the O-rings were dabbed onto *Legionella* selective media (BCYE) and chocolate agar, the plates incubated and bacterial growth identified by matrix-assisted laser desorption/ionization-time of flight mass spectrometry and conventional bacteriological methods. The showerhead parts were rinsed with sterile 0.08% NaCl solution and the washings used to recover *Legionella* species and free-living amoebae.

The contents of showerheads, debris from a thermal mixing valve, fresh and preUV treated water, showerheads, air conditioners and mixer taps from cabins were all cultured for Legionella on selective agar.

Twenty-five showerheads were placed in a stomacher bag and rinsed through with 30 mL of 0.1% peptone water, sealed and shaken for 10 s. The fluid was aspirated and cultured according to AS 3896:2008 using neat and 10^{-1} dilutions of untreated, heat-treated and acid-treated samples onto MWY and BMPAα agars. Plates were incubated in a humid atmosphere at 36 ± 2°C for 7 days with confirmation of presumptive *Legionella* colonies using Oxoid's

Legionella Latex Agglutination test kit. Heterotrophic plate counts were also performed using serial dilutions 10^{-2} to 10^{-5} and incubated at 37°C, as per AS 4276.3.1:2007.

• 7 water samples were processed, as per AS 3896:2008 and AS 4276.3.1:2007 (serial dilutions to 10^{3}) using the contents of showerheads (variable volumes), debris from a thermal mixing valve and fresh and preUV treated water supplies.

• 10 swabs of showerheads, air conditioners, and mixer taps from cabins were cultured with the same methods after the addition of 15 mL of sterile 0.1% peptone.

• 6 shower O-rings were cultured for the presence of thermophilic amoebae by an in-house National Association of Testing Authorities, Australia accredited method using *E.coli* seeded non-nutrient agar plates.

• Presumptive *Legionella* cultures on MWY and BMPAα agars were identified with *Legionella* Latex Agglutination antisera (Thermo Fisher, East Grinstead, UK).

Part 2: Detailed Results

PCR Assay Results (Cycle Thresholds)

Legionella pneumophila PCR was positive in 7/10 cabins where specimens were collected (13/27 samples) (Technical Appendix Table 2). PCR was positive in showerheads or residual water from hand-basin mixer faucets in the cabins of 2 LD cases. In 5 other cabins, only faucets were positive (Technical Appendix Figure 1). Detection of sludge or biofilm in the showerheads and mixer taps prompted recommendation of replacement with a better-designed showerhead and removal of mixer tap aerators.

PCR positive results were all from water outlets. No PCR or culture evidence of Legionella was obtained from upstream hot or cold water supplies.

Only 2/79 samples collected on the second visit were Legionella PCR positive; a significant reduction. (χ^2, Yates' correction; 15.98, $p < 0.001$).

The third series of environmental samples tested on board the vessel found only one of 58 samples was clearly PCR positive, from a tap in a cabin with no connection to LD cases. The in-field PCR results were identical to the confirmatory reference laboratory replicate results.

Reference laboratory PCR assays for *L. pneumophila* produced unambiguous positives in 13/16 showerheads (19/32 samples). Almost all O-rings from the common shower types were *L. pneumophila* positive.

Culture Results

Legionella *Legionella pneumophila* was not isolated from any of the sites sampled or any air samples. Showerhead swabs and agar O ring impressions grew profuse mixed bacteria, commonly *Pseudomonas aeruginosa*. A nonpneumophila *Legionella* sp. was isolated from one showerhead.

Thermophilic amoebae (not *Acanthamoeba* or *Naegleria* spp.) were cultured from 2 shower O-rings.

References

1. World Health Organization. Handbook for inspection of ships and issuance of ship sanitation certificates. Geneva: 2011.

2. World Health Organization. Handbook for management of public health events on board ships. International Health Regulations (2005), Geneva, 2016.

3. Lindsay DS, Abraham WH, Fallon RJ. Detection of mip gene by PCR for diagnosis of Legionnaires' disease. J Clin Microbiol. 1994;32:3068–9. PubMed

4. Chidlow GR, Harnett GB, Shellam GR, Smith DW. An economical tandem multiplex real-time PCR technique for the detection of a comprehensive range of respiratory pathogens. Viruses. 2009;1:42–56. PubMed http://dx.doi.org/10.3390/v1010042

5. Inglis TJ, Merritt A, Montgomery J, Jayasinghe I, Thevanesam V, McInnes R. Deployable laboratory response to emergence of melioidosis in central Sri Lanka. J Clin Microbiol. 2008;46:3479–81. PubMed http://dx.doi.org/10.1128/JCM.01254-08

6. Inglis TJ, Merritt AJ, Levy A, Vietheer P, Bradbury R, Scholler A, et al. Deployable laboratory response to influenza pandemic; PCR assay field trials and comparison with reference methods. PLoS One. 2011;6:e25526. PubMed http://dx.doi.org/10.1371/journal.pone.0025526

7. Inglis TJ, Bradbury RS, McInnes RL, Frances SP, Merritt AJ, Levy A, et al. Deployable molecular detection of arboviruses in the Australian outback. Am J Trop Med Hyg. 2016;95:633–8. PubMed http://dx.doi.org/10.4269/ajtmh.15-0878

8. Inglis TJ. Adapting the mobile laboratory to the changing needs of the Ebolavirus epidemic. J Med Microbiol. 2015;64:587–91. PubMed http://dx.doi.org/10.1099/jmm.0.000063

9. Inglis TJ. The lab without walls: a deployable approach to tropical infectious diseases. Am J Trop Med Hyg. 2013;88:614–8. PubMed http://dx.doi.org/10.4269/ajtmh.12-0704

Technical Appendix Table 1. Results of culture of environmental samples collected on a merchant vessel near the northern coast of Australia, August 27, 2015*

Location, sample	BMPA*	HBA*	Identification
Air sampler control	No growth	No growth	Not applicable
Rm*18, cabin	No growth	1 CFU	*Staphylococcus hominis*
Rm18, shower head	No growth	8 CFU	*Pseudomonas aeruginosa, Staphylococcus hominis, St. epidermidis, St. cohnii*
Rm22, cabin	No growth	7 CFU	*Microbacterium oxidans, St. haemolyticus, Roseomonas* sp.
Rm22, shower head	No growth	10 CFU	*Microbacterium* sp.
Rm34, cabin	No growth	No growth	*Pantoea* sp.
Rm34, shower head	No growth	5 CFU	*St. hominis*
Air Handling Unit	No growth	1 CFU	*Acinetobacter junii*
Galley	No growth	3 CFU	No reliable identification
Rm18, showerhead liquid	No growth	+++	*Pseudomonas aeruginosa*
Rm18, showerhead swab	No growth	++	*Pseudomonas aeruginosa*
Rm18, faucet aerator insert missing, PCR swabs only	Not applicable	Not applicable	Not applicable
Rm22, faucet swab	No growth	++	*Comamonas testeroni*
Rm22, showerhead	No growth	+	*Pseudomonas aeruginosa*
Rm22, showerhead liquid	No growth	+	*Pseudomonas aeruginosa*
Rm04, faucet	No growth	++	*Enterococcus faecalis*

*BMPAα, Buffered Charcoal Yeast Extract Agar alpha medium with antimicrobial agents; HBA, hemolyzed blood agar; Rm, room; + to +++ semi-quantiative positive growth

Technical Appendix Table 2. Results of *Legionella* species PCR assays

Location	CT*	CT	Repeat CT	Repeat CT	Interpretation
Water, Rm*22 faucet	33.32	32.87	NA	NA	Detected
Water, Rm22 showerhead content	34.11	35.71	NA	NA	Detected
Water, Rm18 showerhead	33.56	35.68	NA	NA	Detected
Water, Rm34 showerhead content	–	–	NA	NA	Not detected
Water, post UV, engineer room	–	–	NA	NA	Not detected
Negative control	–	–	NA	NA	Not detected
Water, pre UV, engineer room	–	–	NA	NA	Not detected
Water, washing sink, galley	–	–	NA	NA	Not detected
Swab, Rm34 showerhead	–	–	NA	NA	Not detected
Swab, Rm38, faucet, no diffuser	35.55	33.87	NA	NA	Detected
Swab, Rm30	32.02	32.03	NA	NA	Detected
Negative control	–	–	NA	NA	Not detected
Swab, Rm24 faucet, no diffuser	–	–	NA	NA	Not detected
Swab, Rm22 faucet interior	30.78	30.09	NA	NA	Detected
Swab, Rm22 faucet interior	30.87	30.82	NA	NA	Detected
Swab, Rm20 faucet	32.94	32.73	NA	NA	Detected
Swab, Rm06 faucet	35.32	35.84	NA	NA	Detected
Negative control	–	–	NA	NA	Not detected
Swab, Rm05 faucet, Officer	32.94	33.16	NA	NA	Detected
Swab, Rm04 faucet	–	–	NA	NA	Not detected
Swab, Rm01 faucet Captain	35.29	34.61	NA	NA	Detected
Swab, air handling unit room, chiller†	–	–	NA	NA	Not detected
Swab, Rm18 showerhead	35.61	–	–	–	Not detected
Negative control	–	–	NA	NA	Not detected
Swab, Rm18 faucet	35.8	34.72	NA	NA	Detected
Swab, Rm18 air conditioner inlet	–	–	NA	NA	Not detected

Location	CT*	CT	Repeat CT	Repeat CT	Interpretation
Swab, Rm22 showerhead inner	32.29	32.2	NA	NA	Detected
Swab, Rm22 air con outlet	–	–	NA	NA	Not detected
Swab, Rm22 faucet	–	35.86	–	–	Not detected
Negative control	–	–	NA	NA	Not detected
Swab, Rm34 air conditioner	–	–	NA	NA	Not detected
Swab, Rm34 faucet	–	–	NA	NA	Not detected
Negative control	–	–	NA	NA	Not detected
Positive control	30.11	31.37	NA	NA	Detected
Positive control	31.05	31.52	NA	NA	Detected
Swab, Rm06 toilet tap	–	–	NA	NA	Not detected
Swab, Rm22 distal hose	–	–	NA	NA	Not detected
Swab, Rm16 faucet	–	–	NA	NA	Not detected
Swab, Rm18 faucet	–	–	NA	NA	Not detected
Swab, Rm26 distal hose	–	–	NA	NA	Not detected
Negative control	–	–	NA	NA	Not detected
Swab, Rm14 faucet	–	–	NA	NA	Not detected
Swab, Rm30 faucet	–	–	NA	NA	Not detected
Swab, Rm17 distal hose	–	–	NA	NA	Not detected
Swab, Rm18 distal hose	–	–	NA	NA	Not detected
Swab, Rm06 faucet	–	–	NA	NA	Not detected
Negative control	–	–	NA	NA	Not detected
Swab, Rm20 distal hose	–	–	NA	NA	Not detected
Swab, Rm26 faucet	–	–	NA	NA	Not detected
Swab, Rm07 faucet	–	–	NA	NA	Not detected
Swab, Galley kitchen washing sink	–	–	NA	NA	Not detected
Swab, Rm17 faucet	–	–	NA	NA	Not detected
Negative control	–	–	NA	NA	Not detected
Swab, Rm22 Tap	–	–	NA	NA	Not detected
Swab, Aft public toilet faucet	–	–	NA	NA	Not detected
Swab, Rm28 distal hose	–	–	NA	NA	Not detected
Swab, Rm06 distal hose	–	–	NA	NA	Not detected
Swab, Rm10 Tap #2	–	–	NA	NA	Not detected
Negative control	–	–	NA	NA	Not detected
Swab, Rm24 distal hose	–	–	NA	NA	Not detected
Swab, Rm16 distal hose	–	–	NA	NA	Not detected
Swab, Rm18 showerhead	–	–	NA	NA	Not detected
Swab, Rm21 faucet	–	–	NA	NA	Not detected
Swab, Rm34 faucet	–	–	NA	NA	Not detected
Negative control	–	–	NA	NA	Not detected
Swab, Rm29 faucet	–	–	NA	NA	Not detected
Swab, Rm30 distal hose	–	–	NA	NA	Not detected
Swab, Rm31 distal hose	–	–	NA	NA	Not detected
Swab, Rm28 faucet	–	–	NA	NA	Not detected
Swab, Rm10 faucet #1	–	–	NA	NA	Not detected
Negative control	–	–	NA	NA	Not detected
Swab, Rm02 distal hose	–	–	NA	NA	Not detected
Swab, Rm03 distal hose	–	–	NA	NA	Not detected
Swab, Rm03 faucet	–	–	NA	NA	Not detected
Swab, Rm20 faucet	–	–	NA	NA	Not detected
Swab, Rm14 distal hose	–	–	NA	NA	Not detected
Negative control	–	–	NA	NA	Not detected
Swab, Rm24 faucet	–	–	NA	NA	Not detected
Swab, Rm05 faucet	–	–	NA	NA	Not detected
Swab, Rm05 showerhead	–	–	NA	NA	Not detected
Swab, Rm25 faucet	–	36.46	–	–	Not detected
Swab, Rm19 distal hose	–	–	NA	NA	Not detected
Negative control	–	–	NA	NA	Not detected
Swab, Rm27 faucet	44.02	–	–	35.72	Reproducible discrepant detection
Swab, Laundry sink faucet	–	–	NA	NA	Not detected
Swab, Aft public toilet distal hose	–	–	NA	NA	Not detected
Swab, Rm33 faucet	–	–	NA	NA	Not detected
Swab, Rm10 showerhead	–	–	NA	NA	Not detected
Negative control	–	–	NA	NA	Not detected
Swab, Rm01 faucet	–	–	NA	NA	Not detected
Swab, Rm23 distal hose	–	–	NA	NA	Not detected
Swab, Rm19 faucet	–	–	NA	NA	Not detected
Swab, Rm23 faucet	–	–	NA	NA	Not detected
Swab, Rm27 distal hose	–	–	NA	NA	Not detected
Negative control	–	–	NA	NA	Not detected
Swab, Emergency eye washer	–	–	NA	NA	Not detected
Swab, Rm29 distal hose	–	–	NA	NA	Not detected
Swab, Rm29 distal hose	–	–	NA	NA	Not detected
Swab, Rm04 distal hose	–	–	NA	NA	Not detected

Location	CT*	CT	Repeat CT	Repeat CT	Interpretation
Swab, Rm32 distal hose	–	–	NA	NA	Not detected
Negative control	–	–	NA	NA	Not detected
Swab, Kitchen high pressure no diffuser	–	–	NA	NA	Not detected
Swab, Rm21 distal hose	–	–	NA	NA	Not detected
Swab, Emergency outdoor shower	–	–	NA	NA	Not detected
Swab, Rm31 faucet	–	–	NA	NA	Not detected
Swab, Rm03 Showerhead	–	–	NA	NA	Not detected
Negative control	–	–	NA	NA	Not detected
Swab, Kitchen high pressure sink	–	–	NA	NA	Not detected
Swab, High pressure hose	–	36.42	–	35.12	Discrepant detection
Swab, Rm05 distal hose	–	–	NA	NA	Not detected
Swab, Rm32 faucet	–	–	NA	NA	Not detected
Swab, Kitchen sink no diffuser #2	–	–			Not detected
Negative control	–	–	NA	NA	Not detected
Swab, Rm25 distal hose	–	–	NA	NA	Not detected
Swab, Rm15 faucet	–	–	NA	NA	Not detected
Swab, Rm02 faucet	–	–	NA	NA	Not detected
Swab, Rm22 showerhead	–	–	NA	NA	Not detected
Swab, Rm01 distal hose	–	–	NA	NA	Not detected
Negative control	–	–	NA	NA	Not detected
Swab, Rm15 faucet	–	–	NA	NA	Not detected
Swab, Rm04 faucet	–	–	NA	NA	Not detected
Swab, Rm10 distal hose	–	–	NA	NA	Not detected
Swab, Rm13 distal hose	–	–	NA	NA	Not detected
Swab, Rm34 Showerhead	–	–	NA	NA	Not detected
Negative control	–	–	NA	NA	Not detected
Swab, Rm33 distal hose	–	–	NA	NA	Not detected
Swab, Rm34 distal hose	–	–	NA	NA	Not detected
Swab, Rm07 distal hose	–	–	NA	NA	Not detected
Swab, Rm13 Tap	–	–	NA	NA	Not detected
Negative control	–	–	NA	NA	Not detected
Positive control	32.73	31.98	32.24	30.92	Detected
Positive control	31.94	31.34	31.41	30.02	Detected
Swab, Rm15 faucet	–	–	NA	NA	Not detected
Swab, Rm24 shower	–	–	NA	NA	Not detected
Swab, Rm19 shower	–	–	NA	NA	Not detected
Swab, Rm24 faucet	–	–	NA	NA	Not detected
Swab, Rm26 faucet	36.8	–	–	–	Not detected
Negative control	–	–	NA	NA	Not detected
Swab, Rm17 shower	–	–	NA	NA	Not detected
Swab, Rm06 shower	–	–	NA	NA	Not detected
Swab, Rm26 shower	–	–	NA	NA	Not detected
Swab, Rm28 shower	–	–	NA	NA	Not detected
Swab, Rm23 shower	–	–	NA	NA	Not detected
Negative control	–	–	NA	NA	Not detected
Swab, Rm31 shower	–	–	NA	NA	Not detected
Swab, Rm17 faucet	–	–	NA	NA	Not detected
Swab, Rm27 faucet	–	–	NA	NA	Not detected
Swab, Rm25 shower	–	–	NA	NA	Not detected
Swab, Rm19 faucet	–	–	NA	NA	Not detected
Negative control	–	–	NA	NA	Not detected
Swab, Rm23 faucet	–	–	NA	NA	Not detected
Swab, Rm32 Shower	–	–	NA	NA	Not detected
Swab, Rm25 faucet	–	–	NA	NA	Not detected
Swab, Rm07 shower	–	–	NA	NA	Not detected
Swab, Rm07 faucet	–	–	NA	NA	Not detected
Negative control	–	–	NA	NA	Not detected
Swab, Rm28 faucet	–	–	NA	NA	Not detected
Swab, Rm32 faucet	–	–	NA	NA	Not detected
Swab, Rm18 shower	–	–	NA	NA	Not detected
Swab, Rm06 faucet	–	–	NA	NA	Not detected
Swab, Rm02 shower	–	–	NA	NA	Not detected
Negative control	–	–	NA	NA	Not detected
Swab, Rm34 shower	–	–	NA	NA	Not detected
Swab, Rm29 shower	–	–	NA	NA	Not detected
Swab, Rm22 faucet	–	–	–	–	Inhibitory
Swab, Rm30 shower	–	–	NA	NA	Not detected
Swab, Rm29 faucet	–	–	NA	NA	Not detected
Negative control	–	–	NA	NA	Not detected
Swab, Rm21 shower	–	–	NA	NA	Not detected
Swab, Rm31 faucet	–	35.19	37	36	Detected
Swab, Rm02 faucet	–	–	NA	NA	Not detected
Swab, Rm34 faucet	–	–	NA	NA	Not detected

Location	CT*	CT	Repeat CT	Repeat CT	Interpretation
Swab, Rm22 shower	–	–	NA	NA	Not detected
Negative control	–	–	NA	NA	Not detected
Swab, Rm27 shower	–	–	NA	NA	Not detected
Swab, Rm21 faucet	–	–	NA	NA	Not detected
Swab, Rm30 faucet	–	–	NA	NA	Not detected
Swab, Rm15 shower	–	–	NA	NA	Not detected
Swab, Rm16 faucet	–	–	NA	NA	Not detected
Negative control	–	–	NA	NA	Not detected
Swab, Rm33 faucet	–	–	NA	NA	Not detected
Swab, Rm20 shower	–	–	NA	NA	Not detected
Swab, Rm30 faucet	–	–	NA	NA	Not detected
Swab, Rm13 shower	–	–	NA	NA	Not detected
Swab, Rm15 faucet	–	–	NA	NA	Not detected
Negative control	–	–	NA	NA	Not detected
Swab, Rm05 shower	–	–	NA	NA	Not detected
Swab, Rm01 shower	–	–	NA	NA	Not detected
Swab, Rm04 faucet	–	–	NA	NA	Not detected
Swab, Rm33 shower	–	–	NA	NA	Not detected
Swab, Rm04 shower	–	–	NA	NA	Not detected
Negative control	–	–	NA	NA	Not detected
Swab, Rm16 shower	–	–	NA	NA	Not detected
Swab, Rm14 tap	–	–	NA	NA	Not detected
Swab, Rm13 tap	–	–	NA	NA	Not detected
Swab, Rm03 tap	–	–	NA	NA	Not detected
Swab, Rm03 shower	–	–	NA	NA	Not detected
Negative control	–	–	NA	NA	Not detected
Swab, Rm14 shower	–	–	NA	NA	Not detected
Swab, Rm05 tap	–	–	NA	NA	Not detected
Swab, Rm01 tap	–	–	NA	NA	Not detected
Negative control	–	–	NA	NA	Not detected
Positive control	33.62	32.42	NA	NA	Detected
Positive control	33.4	30.92	NA	NA	Detected
Swab, Rm04 shower	–	–	NA	NA	Not detected
Swab, Rm04 tap	–	–	NA	NA	Not detected
Swab, Rm03 shower	–	–	NA	NA	Not detected
Swab, Rm03 tap	–	–	NA	NA	Not detected
Swab, Rm02 shower	–	–	NA	NA	Not detected
Negative control	–	–	NA	NA	Not detected
Swab, Rm02 tap	–	–	NA	NA	Not detected
Swab, Rm01 shower	–	–	NA	NA	Not detected
Swab, Rm01 tap	–	–	NA	NA	Not detected
Swab, Rm13 shower	–	–	NA	NA	Not detected
Swab, Rm13 tap	–	–	NA	NA	Not detected
Negative control	–	–	NA	NA	Not detected
Swab, Rm07 shower	–	–	NA	NA	Not detected
Swab, Rm07 tap	–	–	NA	NA	Not detected
Swab, Rm06 shower	–	–	NA	NA	Not detected
Swab, Rm06 tap	–	–	NA	NA	Not detected
Swab, Rm05 shower	–	–	NA	NA	Not detected
Negative control	–	–	NA	NA	Not detected
Swab, Rm05 tap	–	–	NA	NA	Not detected
Swab, Rm33 tap	–	–	NA	NA	Not detected
Swab, Rm20 shower	–	–	NA	NA	Not detected
Swab, Rm20 tap	–	–	NA	NA	Not detected
Swab, Rm22 shower	–	–	NA	NA	Not detected
Negative control	–	–	NA	NA	Not detected
Swab, Rm33 shower	–	–	NA	NA	Not detected
Swab, Rm22 tap	–	–	NA	NA	Not detected
Swab, Rm18 shower	–	–	NA	NA	Not detected
Swab, Rm18 tap	–	–	NA	NA	Not detected
Positive control	–	–	NA	NA	Not detected
Positive control	34.35	32.62	NA	NA	Detected

*CT,cutting threshold; rpt, repeat ;Rm, room; NA, not applicable.
†Ship-wide air conditioning machinery.
